# Supplementary material for: Association of Human Cytomegalovirus exposure with tuberculosis disease in South African adults with presumptive tuberculosis
Source: PLOS Glob Public Health. 2026 May 19;6(5):e0006001. doi: 10.1371/journal.pgph.0006001 (PMC13186327; doi:10.1371/journal.pgph.0006001)

**Association of Human Cytomegalovirus exposure with tuberculosis disease in South African adults with presumptive tuberculosis**

Derrick Semugenze, Arthur Chiwaya, George William Kasule, James Sserubiri, Rose Magala, Byron W P Reeve, Zaida Palmer, Hridesh Mishra, Robin Warren, Achilles Katamba, Alberto García-Basteiro, Moses L Joloba, Grant Theron, Frank Cobelens, Willy Ssengooba

**Table of Contents**

[Effect Modification Analysis Between HCMV DNAemia and BMI on TB Disease 2](#_Toc229075944)

[Mediation Analysis of the Effect of HCMV DNAemia on TB disease through Hemoglobin (Hb) 3](#_Toc229075945)

[Relationship between HCMV viral load and Time to Positivity (TTP) for Mycobacterium cultures 5](#_Toc229075946)

[Distribution of HCMV viral load stratified by mycobacterial culture results among HIV-positive participants 6](#_Toc229075947)

[Distribution of HCMV DNAemia viral load stratified by prior TB disease history 7](#_Toc229075948)

[Distribution of HCMV DNAemia viral load by tuberculosis (TB) disease classification 8](#_Toc229075949)

**List of figures**

[**Fig A.** DAG showing the hypothesized causal relationship among HCMV, TB and other factors 3](#_Toc211507095)

[**Fig B.** Scatter plot showing variation between HCMV viral load and TTP 6](#_Toc211507096)

[**Fig C.** Boxplot showing the distribution of HCMV viral load stratified by TB disease state 7](#_Toc211507097)

[**Fig D.** Scatter plot illustrating individual HCMV DNAemia viral load (copies/mL) by previous TB disease status. 8](#_Toc211507098)

[**Fig E.** HCMV viral load by TB disease classification among participants. 9](#_Toc211507099)

**List of tables**

[**Table A.** BMI category modification of the association between HCMV DNAemia and TB disease 3](#_Toc211507109)

[**Table B.** Causal mediation analysis of Hemoglobin in the association between HCMV DNAemia and TB disease 4](#_Toc211507110)

[**Table C.** Distribution of study participants by HCMV exposure category 5](#_Toc211507111)

**Directed Acyclic Graph illustrating hypothesized causal relationships between human cytomegalovirus (HCMV) exposure and tuberculosis (TB) disease**

The Directed Acyclic Graph (DAG) shown in Figure 1 represents the hypothesized causal relationships among HCMV exposure, TB disease, and several associated covariates considered in this study. HCMV exposure, the primary variable of interest, is positioned as a direct cause of TB disease. Potential confounders, including HIV status, smoking, race, and gender (illustrated as red nodes) are modeled as common causes influencing both HCMV exposure and TB disease, reflecting their capacity to introduce bias in naive exposure-outcome associations. BMI is also treated as a confounder affecting both the exposure and outcome and an effect modifier. Hemoglobin acts as a potential mediator on the causal path between HCMV exposure and TB disease, symbolizing the biological mechanisms through which HCMV infection could affect TB risk via alterations in hemoglobin levels. The directional arrows represent assumed causal influences based on epidemiological theory and prior evidence. Black arrows indicate direct causal pathways, the pink arrows indicate alternative influences or mediating processes and the green one indicates the hypothesized pathway.


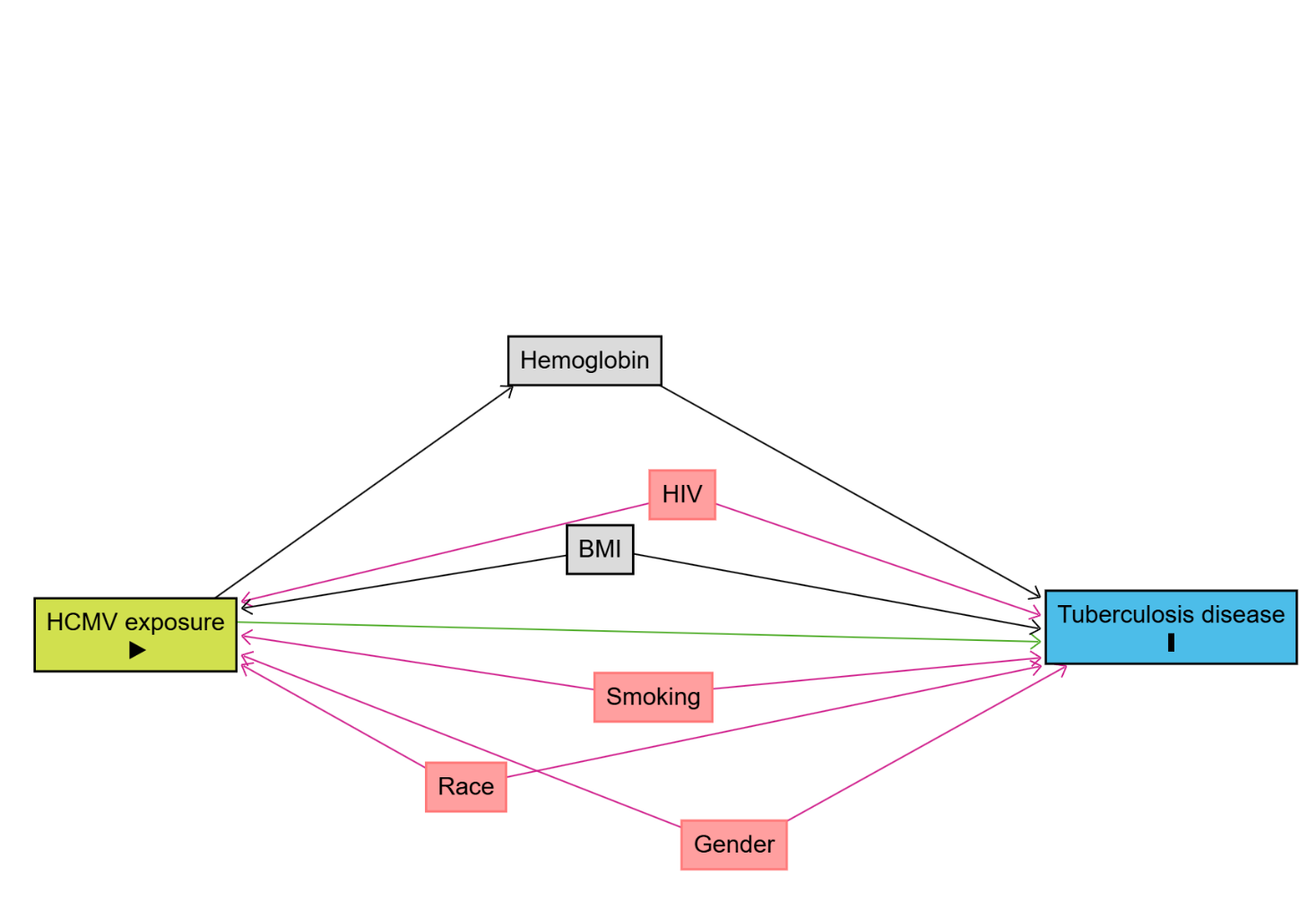


**Fig A:** DAG showing the hypothesized causal relationship among HCMV, TB and other factors

# **Effect Modification Analysis Between HCMV DNAemia and BMI on TB Disease**

This table presents an effect modification analysis investigating how body mass index (BMI) categories modify the association between HCMV DNAemia and TB disease. The model includes main effects for HCMV DNAemia, BMI categories (underweight, overweight), age groups, gender, and HIV status, along with interaction terms between HCMV DNAemia and BMI categories. Odds ratios (ORs), standard errors, test statistics, p-values, and 95% confidence intervals (CIs) are provided for each term.

**Table A.** BMI category modification of the association between HCMV DNAemia and TB disease

| Term | OR | Std. Error | Statistic | P-value | 95% CI Lower | 95% CI Upper |
| --- | --- | --- | --- | --- | --- | --- |
| (Intercept) | 1.13 | 0.29 | 0.42 | 0.670 | 0.64 | 2.01 |
| HCMV DNAemia | 2.84 | 0.75 | 1.39 | 0.178 | 0.61 | 12.61 |
| Underweight | 2.03 | 0.31 | 2.31 | 0.018 | 1.11 | 3.71 |
| Overweight | 0.15 | 0.53 | -3.58 | <0.001 | 0.05 | 0.4 |
| 36-50 years | 0.63 | 0.32 | -1.46 | 0.143 | 0.33 | 1.17 |
| >50 years | 0.51 | 0.39 | -1.74 | 0.082 | 0.23 | 1.07 |
| Male | 0.45 | 0.29 | -2.7 | 0.007 | 0.25 | 0.8 |
| HIV positive | 0.59 | 0.32 | -1.64 | 0.139 | 0.31 | 1.1 |
| HCMV DNAemia x Underweight | 4.01 | 1.32 | 1.05 | 0.297 | 0.38 | 100.03 |
| HCMV DNAemia x Overweight | 4.92 | 1.52 | 1.05 | 0.298 | 0.16 | 92.28 |

# **Mediation Analysis of the Effect of HCMV DNAemia on TB disease through Hemoglobin (Hb)**

This table presents the different parameters that show how hemoglobin mediates the association between HCMV DNAemia and TB disease. The ACME is the indirect effect of HCMV exposure on TB disease through hemoglobin. The ADE is the direct effect of HCMV exposure on TB disease, not through hemoglobin. The total effect is the overall effect (both direct and mediated) of HCMV exposure on TB disease. The Proportion Mediated is the fraction of the total effect that is explained by the mediator (hemoglobin). The control indicates the analyzed parameters in individuals without HCMV DNAemia while the treated indicates the observed ones in individuals with HCMV DNAemia. The average indicates the mean across all participants.

**Table B.** Causal mediation analysis of Hemoglobin in the association between HCMV DNAemia and TB disease

| Effect | Estimate | 95% CI Lower | 95% CI Upper | P-value |
| --- | --- | --- | --- | --- |
| ACME (control) | 0.066 | 0.006 | 0.144 | 0.032 |
| ACME (treated) | 0.072 | 0.005 | 0.151 | 0.026 |
| ADE (control) | 0.258 | 0.046 | 0.497 | 0.01 |
| ADE (treated) | 0.263 | 0.049 | 0.483 | 0.01 |
| Total Effect | 0.329 | 0.114 | 0.54 | 0.004 |
| Proportion Mediated (control) | 0.201 | 0.013 | 0.617 | 0.032 |
| Proportion Mediated (treated) | 0.217 | 0.017 | 0.649 | 0.026 |
| ACME (average) | 0.069 | 0.005 | 0.147 | 0.028 |
| ADE (average) | 0.261 | 0.047 | 0.492 | 0.01 |
| Proportion mediated (average) | 0.209 | 0.010 | 0.634 | 0.028 |

N = 291, 1000 bootstrap simulations

ACME = Average Causal Mediation Effect

ADE = Average Direct Effect

Estimates of Average Causal Mediation Effect (ACME), Average Direct Effect (ADE), total effect, and proportion mediated are presented for control and treated groups, including 95% confidence intervals and p-values. The analysis used 1000 bootstrap simulations on a sample of 291 participants.

**Numbers of cases and controls under the different HCMV exposure categories**

Only 2 participants had primary HCMV infection as indicated by low anti-HCMV IgG avidity: 1 case (1.0%) and 1 control (0.5%). Current HCMV reactivation or reinfection was observed in 12 (57.1%) cases and 9 (42.9%) control, recent HCMV reactivation or reinfection in 6 (33.3%) cases and 11 (66.7%) control, and Past HCMV infection in 80 cases (31.0%) and 178 controls (69.0%).

**Table C.** Distribution of study participants by HCMV exposure category

| HCMV exposure category | Description | Cases | Controls | All participants (%) |
| --- | --- | --- | --- | --- |
| Primary HCMV infection | Participants with low anti-HCMV IgG avidity irrespective of HCMV DNAemia and anti-HCMV IgM results | 1 | 1 | 2 (1%) |
| Current HCMV reactivation or reinfection | Participants with HCMV DNAemia, and negative or positive for anti-HCMV IgM who had high anti-HCMV IgG avidity | 12 | 9 | 21 (7.1%) |
| Recent HCMV reactivation or reinfection | Participants without HCMV DNAemia, and positive for anti-HCMV IgM with high anti-HCMV IgG avidity | 5 | 11 | 16 (5.4%) |
| Past HCMV infection | Participants without HCMV DNAemia and; negative for anti-HCMV IgM and with high anti-HCMV IgG avidity | 80 | 178 | 258 (86.9%) |
| Total | | **98** | **199** | **297** |

# **Relationship between HCMV viral load and Time to Positivity (TTP) for Mycobacterium cultures**

This scatter plot illustrates the relationship between HCMV viral load and Time to TB Positivity (TTP). Each dot represents an individual participant’s paired measurement of viral load and TTP. The red dashed line shows the fitted regression line, which summarizes the overall trend between the two variables. The gray shaded band represents the 95% confidence interval, indicating the range within which the true regression line is likely to fall with 95% certainty.

**Fig B.** Scatter plot showing variation between HCMV viral load and TTP


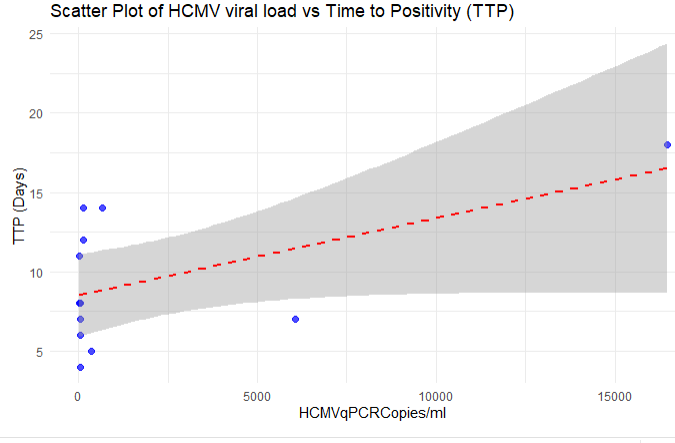


HCMVqPCRCopies = HCMV viral load in copies/ml

TTP (Days) = Time to TB Positivity (TTP) in days

# **Distribution of HCMV viral load stratified by mycobacterial culture results among HIV-positive participants**

Data points superimposed on the boxplots represent individual observations, with the red box indicating the interquartile range and median viral load for cases (TB culture and or GeneXpert MTB/Rif positive), while the bluebox displays the distribution for controls (negative for culture and GeneXpert MTB/Rif).

**Fig C.** Boxplot showing the distribution of HCMV viral load stratified by TB disease state


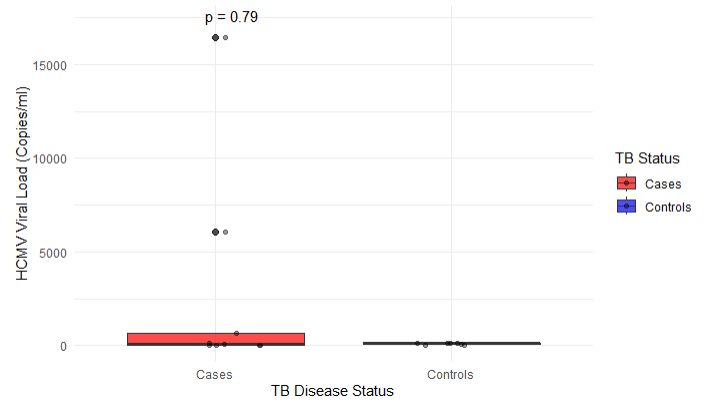


# **Distribution of HCMV DNAemia viral load stratified by prior TB disease history**

This scatter plot depicts the distribution of HCMV DNAemia viral load, measured as copies per milliliter, stratified by prior tuberculosis (TB) disease history. On the x-axis, participants are categorized into those with and without a previous history of TB. The y-axis shows individual HCMV DNAemia viral load values.

**Fig D.** Scatter plot illustrating individual HCMV DNAemia viral load (copies/mL) by previous TB disease status.


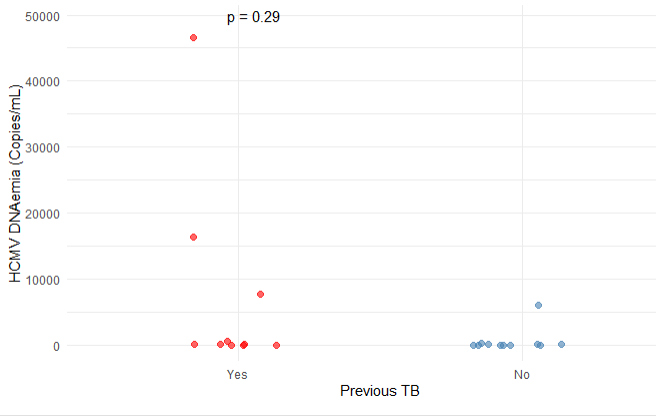


# **Distribution of HCMV DNAemia viral load by tuberculosis (TB) disease classification**

HCMV viral load was compared across two categories of TB disease: TB cases with prior TB treatment and TB cases without previous TB disease. While most participants in both groups exhibited low or undetectable HCMV DNAemia, some individuals demonstrated markedly higher viral loads, with peak values exceeding 15,000 copies/ml, particularly among TB cases with prior TB treatment.

**Fig E.** HCMV viral load by TB disease classification among participants.


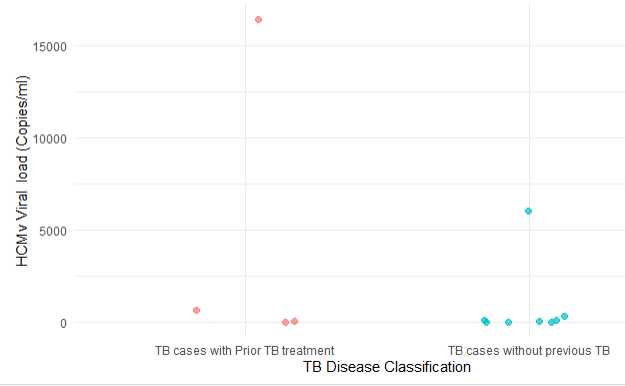

Supplement: S1 Text — (DOCX) [file pgph.0006001.s002.docx]
