# Supplementary material for: Association of Human Cytomegalovirus exposure with tuberculosis disease in South African adults with presumptive tuberculosis
Source: PLOS Glob Public Health. 2026 May 19;6(5):e0006001. doi: 10.1371/journal.pgph.0006001 (PMC13186327; doi:10.1371/journal.pgph.0006001)
Supplement: Attachment — Submitted filename: Response to Reviewers`.docx [file pgph.0006001.s003.docx]

**RE: POINT-BY-POINT RESPONSES TO THE REVIEWER`S COMMENTS MANUSCRIPT “SUBMISSION ID: PGPH-D-26-00309”**

Dear Academic Editor,

PLOS Global Public Health

Thank you for this opportunity to submit a revised version of our manuscript and respond to the reviewers' comments. We greatly appreciate the review comments and we have done our best to address them.

Please find below our point-by-point response. We have tried as much as possible to show the textual revisions as part of our responses. We do not show changes in the order of sentences or words in sentences, neither do we show words taken out to comply with the word count limit. These revisions can be seen in the marked-up version of the revised manuscript.

Yours sincerely

Dr. Willy Ssengooba, corresponding author

**REVIEWER #1**

Comment: **Thank you for the opportunity to review this work. The research question is relevant and timely. My primary concern relates to the categorization of HCMV exposure in the secondary analyses. The grouping strategy is difficult to follow and, in some instances, combines physiopathologically distinct entities (e.g., primary acute CMV infection with CMV reactivation or reinfection). Furthermore, some categories include very small numbers of participants, which limits interpretability and statistical robustness.**

Comment: **If possible, I suggest simplifying the nomenclature and analytical framework to improve clarity and readability. For example, instead of using the category “current HCMV reactivation or reinfection” consider using “current HCMV infection” with clear explanation in Methods that “current” infection may include new infection, reactivation or reinfection. A similar simplification could be applied to the “recent” categories (e.g. “recent CMV infection” instead of “recent CMV infection or reinfection”).**

**Response:** We acknowledge that our HCMV exposure categorization nomenclature could be simplified. For our secondary analysis we have now collapsed the categories as follows (see revised Box):

“**Primary HCMV infection**: *Participants with low anti-HCMV IgG avidity* irrespective of *HCMV DNAemia and anti-HCMV IgM results*

**Current HCMV infection**: *Participants with HCMV DNAemia and high anti-HCMV IgG avidity irrespective of anti-HCMV IgM results*

**Recent HCMV infection**: *Participants without HCMV DNAemia, and positive for anti-HCMV IgM with high anti-HCMV IgG avidity*

**Past HCMV infection**: *Participants without HCMV DNAemia who tested negative for anti-HCMV IgM and had high anti-HCMV IgG avidity”*

This categorization has now been consistently used throughout the revised manuscript.

ABSTRACT

1. Comment: **Lines 33-37: The sentence contains minor grammatical errors and unclear phrasing, particularly regarding the definition of cases and controls. Please revise for clarity and precision.**

**Response:** The sentence has been revised and currently as “This study analyzed TB Cases (n=98) who were mycobacterial culture and or GeneXpert Ultra positive and controls (n = 199) who were frequency matched by HIV status.”.

2. Comment: **Lines 40-42: Revise. The main results presented in an Abstract should prioritize the primary analysis, such as providing and comparing the proportion of participants positive CMV DNAemia in both study arms.**

**Response:** The primary analysis has now been prioritized.

3. Comment: **Line 42-44: Again, why are the results from the logistic regression for the secondary outcome (combined HCMV serology and DNA results) presented instead of the primary outcome (DNA only)?**

**Response:** We agree with the reviewer’s point. The primary analysis (HCMV DNAemia) has been prioritized.

4. Comment: **Line 43-47: The multiple HCMV categories are difficult to follow in the Abstract and require readers to consult Methods section for interpretation. I suggest simplifying this classification.**

**Response:** The HCMV categories have been simplified as suggested by the reviewer and the sentence is reading as “A similar association was observed for current HCMV infection well as no association with recent HCMV infection was found”

INTRODUCTION:

1. Comment: **Lines 71-72: The statement regarding the evidence for the existence of CMV reinfections (with a genetically distinct strain form the original), does not appear to be directly supported by reference n°10. Please verify and revise the citation accordingly.**

**Response:** The citation has been replaced and the sentence was revised as follows: “Over 90% of adults in developing countries are seropositive for HCMV(ref) and both reactivations of the initial infection and reinfections with new strains occur (ref). In TB endemic countries, adults are likely to have reactivation HCMV or even reinfection HCMV rather than primary disease (ref).”

METHODS

1. Comment: **Lines 82-83: Please clarify: Who are the participants with “non-actionable culture results”? Who are “those with missing” (missing what?) What is meant by “inappropriate” sample? or “missing sample volumes”?**

**Response:** These have been clarified and the sentence in the revised manuscript as reads follows: “Participants without diagnostic testing and those with contaminated cultures without GeneXpert MTB/RIF Ultra results were excluded from analysis.”

2. Comment: **Line 84-87 (Controls): Please clearly define the control group: Were they individuals with presumptive pulmonary TB in whom the diagnosis was ultimately excluded? How were controls selected? The manuscript states they were selected “randomly”, but the procedure requires detailed description to ensure internal validity.**

**Response:** The control group and their selection have been clarified in the revised manuscript as follows: “Controls were randomly selected using a random number generator from presumptive pulmonary TB patients who tested negative for both mycobacterial culture and GeneXpert MTB/RIF Ultra. Controls were selected to achieve approximately a 2:1 control-to-case ratio and were frequency matched to TB cases by HIV status. This resulted in 71 controls living with HIV and 128 controls without HIV infection.”

3. Comment: **Lines 92-98 (HCMV qPCR): Please provide a reference for real-time qPCR method. Was qPCR performed in serum or plasma? Clarify that the primary outcome (HCMV DNAemia) was analyzed qualitatively (positive/ negative).**

**Response:** The references for HCMV qPCR have been provided and we have clarified that plasma was used. We have also indicated that HCMV DNAemia was analyzed qualitatively.

4. Comment: **IgG avidity testing: Please provide the rationale for including HCMV IgG avidity testing. Describe its temporal interpretation in HCMV infection and cite references specific to HCMV IgG avidity.**

**Response:** The rationale for IgG avidity inclusion has now been added to the Statistical analysis subsection as follows: “For primary HCMV infection we required low IgG avidity which indicates primary infection that occurred in previous 3 months while anti-HCMV IgM can be negative; high IgG avidity is generally observed from 2-4 months following primary infection”.

5. Comment: **CMV Exposure Categories (Box): The exposure categories require stronger justification, and ideally supporting references. Each category should be clearly and consistently defined: “Primary CMV Infection”: Given that IgM may persist positive for up to 6 months (Lazzarotto T, et al. J Clin Virol. 2008;41(3):192–197), on what basis were participants with negative IgM but positive IgG with low avidity classified as “primary infection”? Also, how were participants with negative IgG but positive IgM classified? “Recent CMV reactivation or reinfection”: Why was a positive IgM required for this category? Many reinfections and reactivations occur without detectable IgM. “Past CMV infection”: revise the definition, there appears to be a repetition in the final sentence.**

**Response:** With regard to primary CMV infection, we agree that IgM responses may persist for up to 6 months. This is one of the reasons for requiring low IgG avidity to classify a CMV infection as primary. Several authors (e.g. Leruez-Ville and Ville, 2017, <https://doi.org/10.1016/j.bpobgyn.2016.10.005>) have highlighted the usefulness of low IgG avidity in the diagnosis of primary HCMV infection, as well as other shortcomings of using anti-HCMV IgM including transient responses in rare cases, cross-reactivity with other viral agents, and rise in levels due to reactivation. These factors call for cautious interpretation of a positive IgM, hence several authors have argued that low IgG avidity is enough to identify primary infection irrespective of IgM results.

Participants with negative IgG but positive IgM (false negative IgG or false-positive IgM) were not classified as we did not expect them to occur. Indeed, we observed none.

Recent CMV reactivation or reinfection: we required positive IgM for this category because this was the only way in which recent reactivation or reinfection could be observed in the absence of CMV DNAemia.

We acknowledge the limitation that many reinfections and reactivations occur without detectable IgM but a positive IgM is the closest to recent CMV reactivation or reinfection that we could find.

Under Past HCMV infection, the repeated final sentence has been deleted.

The reviewer’s comment made us aware that we could have provided a more extensive justification for our categorization of CMV infection and have now added the following to the Statistical Analysis subsection:“Our secondary analysis investigated the association of TB disease with four categories of HCMV infection based on combining qPCR and serology results (Box). The rationale for this approach was two-fold. First, it allowed us to capture recent active HCMV infection that existed during the months before the patients presented for diagnosis while at that timepoint HCMV DNAemia was no longer present due to diagnostic delay, which for TB is often weeks to months. As IgM and low-avidity IgG responses can persist for months, these responses provide a pre-diagnostic window in which active HCMV has likely been present. Second, it allowed us to distinguish between primary infection versus reactivation or reinfection and explore possible differential associations with TB. For primary HCMV infection we required low IgG avidity which indicates primary infection that occurred in previous 3 months while anti-HCMV IgM can be negative; high IgG avidity is generally observed from 2-4 months following primary infection (ref). For recent HCMV infection (which could be due to reactivation or reinfection) we required a positive HCMV IgM because, despite its limited sensitivity, this was the only way recent infections could be ascertained in our study. Past HCMV infection was considered for all HCMV DNA negative and IgG positive participants that were IgM negative and had high IgG avidity.”

6. Comment: **Lines 142-146: Are these considered secondary outcomes or exploratory analyses? Please clarify.**

**Response:** It has been clarified as follows: “Descriptive exploratory analyses were also conducted (…)”

7. Comment: **Line 144: Please clarify the outcome described as “variation according to previous TB history”.**

**Response:** This sentence has been corrected as follows: “(..) and a history of previous TB disease”.

8. Comment: **Sample size: Please provide sample size calculations for the primary outcome, including assumed effect size, power, alpha, and the intended case-control ratio**

**Response:** We have included the following to the Study design and participant flow subsection: “We expected this sample size to allow us to find a 25% or larger prevalence of HCMV infection among patients diagnosed with TB as significantly (level 0.05) different from an HCMV prevalence among the control group of 5% (power 0.8), allowing for controlling for confounding variables.”

RESULTS

1. Comment: **Characteristics of participants with positive HCMV DNAemia: Please describe the demographic and clinical characteristics (age, sex, HIV status, etc) among CMV DNApositive participants in both study arms. Given the known interaction between HIV and CMV reactivation, I strongly recommend adding a stratified analysis of the primary outcome (HCMV DNAemia and TB disease) by HIV status.**

**Response:** The demographic and clinical characteristics table (Table 1) of the revised manuscript has been reviewed to stratify TB cases and controls by HCMV DNAemia status. HIV status is also among the clinical characteristics that are included in this table. We reflected this in the results section as follows:

“HCMV DNAemia was observed in 12 (12.2%) of cases and 9 (4.5%) controls (Table I). The age distribution differed significantly across groups (p = 0.005). Among the cases, HCMV positivity was more frequent in individuals aged 36–50 years (58.3%), whereas HCMV-negative TB cases were predominantly aged 18–35 years (55.8%). A similar pattern was observed among controls, where 77.8% of HCMV-positive individuals were aged 36–50 years, compared with 37.9% of HCMV-negative controls aged 18-35. Gender distribution did not differ significantly (p = 0.102), although males were more common among HCMV-positive participants, representing 66.7% of TB cases and 88.9% of controls. Body mass index (BMI) categories differed significantly across groups (p<0.001). Underweight status was most common among HCMV-positive TB cases (58.3%), while healthy weight predominated among controls, particularly HCMV-positive controls (62.5%). HIV infection was present in 75.0% of HCMV-positive TB cases and 77.8% of HCMV-positive controls, compared with 29.1% of HCMV-negative TB cases and 33.7% of HCMV-negative controls (p = 0.001) and the overall HIV prevalence was 105 (35.4%).”

Adjusted and interaction analysis has been done between HCMV DNAemia, TB disease and HIV positivity and the description of the results reads as “In a stratified analysis of the HCMV DNAemia and TB disease by HIV status (Table IV), HCMV DNAemia was associated with higher odds of TB disease (OR: 3.20, 95%CI 1.26–8.41; p = 0.015), whereas HIV positivity was not significantly associated with TB disease (OR: 0.84, 95%CI 0.48–1.43; p = 0.53). In the interaction model, the effect of HCMV DNAemia on TB did not differ by HIV status.”

2. Comment: **Line 171: Please provide the proportion of HCMV DNA-positive participants for both groups, not only among TB cases.**

**Response:** The proportion of HCMV DNA-positive participants in both TB cases and controls has been described and it reads as follows “Detection of HCMV DNAemia was observed in 12 (12.2%) of TB cases and 9 (4.5%) controls (Table I).”

3. Comment: **Tables 1 and 2: Provide p-values for baseline group comparisons (including those mentioned in lines 161-169). Also, please present variables consistently (either Yes/No, or Present/Absent, but not both).**

**Response:** The p-values have been included in Tables 1 and 2 on pages 21-23 of the revised manuscript. The variables have now been consistently presented

4. Comment: **Secondary results (from line 185 onward): Please organize results according to the same infection categories presented in the Box and maintain consistent nomenclature throughout.**

**Response:** The results have now been organized according to the same infection categories in the Box and nomenclature is maintained.

5. Comment: **Merging of exposure categories: The category “primary HCMV infection” (N=1 per group) was merged with “recent HCMV reactivation or reinfection,” creating a new category (“recent HCMV infection, reactivation or reinfection”). This is problematic for two reasons: These conditions are physiopathologically distinct. The resulting category is difficult to interpret. I would recommend excluding the primary infection category from analysis rather than merging it.**

**Response:** The “Primary HCMV infection” was excluded from further analyses as suggested. The data henceforth presented in the manuscript and in Table II does not include this category.

6. Comment: **Supplementary. Figures 3 and 4: Please provide statistical significance directly in the figures.**

**Response:** The p-values have been included in the respective figures on pages 7-8 of the revised supplementary data file.

DISCUSSION

1. Comment: **Low hemoglobin as mediator: The hypothesis that anemia may mediate the relationship between CMV and TB is interesting. However, reverse causality should be discussed. Anemia may reflect early, undiagnosed TB rather than a mediator of CMV-driven susceptibility.**

**Response:** Thank you very much for this insightful comment. The reverse causation discussion has been included in the discussion and it reads as “The observed anemia may be due to a reverse causation where it is being caused by either early or undiagnosed tuberculosis disease. Anemia has been a reported comorbid among tuberculosis patients (ref) which has been attributed to inflammation(ref) and there is evidence of reduced anemia with TB treatment without additional iron based interventions(ref)”.

2. Comment: **Biological sampling limitations: Please discuss limitations of measuring HCMV DNAemia in plasma versus whole blood, particularly regarding sensitivity for detecting latent or low-level reactivation.**

**Response:** The limitation of using plasma rather than whole blood has been included in the revised manuscript and it reads as “The use of plasma in diagnosing HCMV DNAemia was reported to miss out some positive participants especially those that had low DNAemia in whole blood (ref) hence we could have missed some participants with latent or low-level reactivation”.

3. Comment: **Lines 216-218: The sentence is difficult to follow and requires revision: “Current HCMV reactivation or reinfection was associated with increased odds of TB disease well as recent HCMV infection, reactivation or reinfection was not.”**

**Response:** The sentence has been revised and it reads as “Unlike recent infection, current HCMV infection was associated with increased odds of TB disease”.

4. Comment: **Interpretation of secondary subgroup analyses: The study appears underpowered for multiple subgroup comparisons. Therefore, statements such as (line 223-227) “These findings suggest that […] recent HCMV infection, reactivation or reinfection plays no or only a limited role in adult TB pathogenesis” remain speculative and should be tempered.**

**Response:** The sentence has been tempered to be more speculative and it reads as “Our findings show that current HCMV infection and TB may be associated when measured at the same time point but showed no association of active TB with earlier HCMV exposure (recent HCMV infection). These findings could suggest that while primary HCMV infection may influence TB susceptibility early in life, recent HCMV infection might play no or only a limited role in adult TB pathogenesis”.

5. Comment: **Temporal argument against causality (Lines 244–252): TB has a prolonged preclinical phase that may extend over several months. The finding that only current—but not recent or prior—CMV reactivation/reinfection is associated with TB disease also weakens a causal interpretation. This issue should be discussed more critically**

**Response:** Effort has been made to discuss the temporal argument against causality more critically and it reads as “The observed association of current HCMV infection with TB as reported in this study may be due to expansion of the CD4+CD28null T cells associated with impaired immune responses to numerous antigens which may include Mtb during the active virus replication phase(ref) This same study reported reduced proportion of this T cell subset during HCMV treatment with valacyclovir which may also indicate that during recent HCMV infection phase, there is lower HCMV antigens hence reduced CD4+CD28null T cells and hence no reported association with TB disease in this group. Tuberculosis disease has a long preclinical phase with subclinical or incipient TB disease lasting for several months to years with a shorter progression period in HIV endemic settings(ref). The TB disease progression may be due to immune dynamics brought about by latent, reactivation and reinfection HCMV cycles over time rather than a single acute/sporadic effect. HCMV reactivation may also be a consequence of TB disease immune activation because the virus is mostly detected from tissues undergoing inflammation(ref). An alternative hypothesis may be that underlying decline in cellular immune functionality with age(ref) which may trigger both progression to TB disease and HCMV reactivation.”.

**REVIEWER #2**

**Comment: Thank you for your submission. Quite an interesting article Please see below some comments.**

**Response: Thank you very much for the comments and taking time to review our manuscript**

**Introduction**

1. **Comment: Please update to 2024 numbers**

**Response: This has been updated**

Methods

2. **Comment: So both cases and controls have presumptive TB with symptoms? Please clarify**

**Response: Yes, both cases and controls were TB presumptive and this is stated in the revised manuscript**

3. **Comment: Seems both a change in estimate and DAG were used for confounder assessment. Can you please explain the logic behind using both especially when you have a DAG?**

**Response: We used a DAG to define which covariates should be treated as potential confounders and which should not. Since numbers of current and recent CMV infections in our analyses were small, we attempted to avoid overfitting by only including those potential confounders that showed a change in estimate.**

**Results**

4. **Comment: Line 165. There is a type. N=297 is overall and not the n for HIV**

**Response: This was rectified in the revised manuscript**

5. **Comment: Line 171. Not sure what the N and % here are referring to. Please clarify**

**Response: This was a mistake during writing. The section has been re-written as follows;”** **Detection of HCMV DNAemia was observed in 12 (12.2%) of TB cases and 9 (4.5%) controls (Table I).”.** The univariate analysis has been properly described as “In a univariate analysis, participants with TB had about three odds of HCMV DNAemia (odds ratio [OR] 2.95, 95% confidence interval [95%CI] 1.20–7.47; p=0.019: Table III).” in the revised manuscript.

6. **Comment: Can you explain the hypothesized pathway for why Hg would be a mediator in this association? How does HCMV lead to change in Hg that then leads to TB?**

**Response:** **This is described under the discussion section and it reads as “Our hypothesis of hemoglobin being a mediator of an HCMV DNAemia-driven susceptibility to TB disease may be true because HCMV infection has been indicated to have direct effects on the bone marrow leading to pancytopenia (ref) as well as causing immune mediated hemolytic anemia (ref). Anemia particularly iron deficiency one has been associated with tuberculosis disease (ref) through causing changes in T cell subsets and cytokine profiles(ref)”.**

7. **Comment: Why was primary merged with recent reactivation/reinfection and not current infection? One would think primary infection would be similar to current re-infection.**

**Response: Due to small numbers and distinct physiopathology in this category, “Primary HCMV infection” has now been excluded from further analyses.**

Discussion

8. **Comment: I would refrain from calling it nested CC. Nested CC may refer to a case control study in a prospective cohort with longitudinal follow up where exposure is collected before the outcome. Here you are doing a cross-sectional analysis with both being assessed at the same time.**

**Response: This has been corrected in the revised manuscript**

9. **Comment: Line 253. This is an interesting finding. If the hypothesis is that TB leads to viral reactivation then low burden of TB bacteria should show low viremia and a dose response (as TB burden increases so does viremia) or higher burden of TB with low viremia. However if viremia leads to TB reactivation, then higher burden of viremia can lead to TB disease even with smaller TB load.**

**Response: While we agree is interesting, we prefer not to over interpret our finding that lower Mtb bacterial load was associated with higher CMV viral load. First, numbers are small, therefore we could not adjust this association for possible confounding. Second, Mtb bacterial burden may not be a direct correlate of inflammation, and thereby not of viral replication if the association would be causal.**

**Figure 1**

10. **Comment: Not sure what randomly selected means as 98 of 99 were selected**

**Response: Only controls were randomly selected. This has been rectified in the revised manuscript**

11. **Comment: Box. "and had high anti-HCMV IgG avidity" is repeated for Past HCMV infection definition**

**Response: Under Past HCMV infection, the repeated final sentence has been deleted.**

**REVIEWER #3**

**Comment: Significance: This study by Semugenze, et al. aims to define the association between recent or active cytomegalovirus (CMV) infection and TB disease. Several prior studies have documented an association between acquired CMV infection and TB risk, particularly in pediatric populations. In adults, the majority of the population that is also susceptible to TB is CMV seropositive, but CMV reactivation may still impact TB disease progression. Controls were matched to TB cases at 2:1 in this South African cohort, which were divided based on recency of CMV infection into: primary HCMV infection, current CMV reactivation/infection, recent CMV reactivation/re-infection and past CMV infection using a combination of qPCR and antibody tests. Current CMV reactivation showed a strong association with TB, which was supported by the data. Overall, this is an important study that strengthens the hypothesis that CMV infection increases TB risk.**

**Response: Thank you very much for this summary of our work**

- **Comment: Can you explain the rationale of combining the two people with primary HCMV infection (one TB case and one control) with the rest of the HCMV reactivation/re-infection group? It would be a better sensitivity analysis to remove these members, especially at such a small sample size.**

**Response: Due to small numbers and distinct physiopathology in this category, “Primary HCMV infection” has now been excluded from further analyses.**

- **Comment: Is there any support in the literature for the idea that TB itself is driving CMV reactivation?**

**Response: We did not come across evidence from literature that supports that TB drives HCMV reactivation, our argument is speculative.** Effort has been made to discuss the temporal argument against causality more critically in the revised manuscript.

- **Comment: Can you speculate reasons for the association between HCMV and longer time to positivity? Are you suggesting that CMV-induced inflammation is associated with faster Mtb control? This seems counterintuitive based on the rest of the data in the paper.**

**Response: While we agree is interesting, we prefer not to over interpret our finding that lower Mtb bacterial load was associated with higher CMV viral load. First, numbers are small, therefore we could not adjust this association for possible confounding. Second, Mtb bacterial burden may not be a direct correlate of inflammation, and thereby not of viral replication if the association would be causal.**
